# Clinical Correlation of Transcription Factor SOX3 in Cancer: Unveiling Its Role in Tumorigenesis

**DOI:** 10.3390/genes15060777

**Published:** 2024-06-13

**Authors:** Helen Lima Del Puerto, Ana Paula G. S. Miranda, Dinah Qutob, Enio Ferreira, Felipe H. S. Silva, Bruna M. Lima, Barbara A. Carvalho, Bruna Roque-Souza, Eduardo Gutseit, Diego C. Castro, Emanuele T. Pozzolini, Nayara O. Duarte, Thacyana B. G. Lopes, Daiana Y. O. Taborda, Stella M. Quirino, Ahmed Elgerbi, John S. Choy, Adam Underwood

**Affiliations:** 1 Department of General Pathology, Universidade Federal de Minas Gerais, Belo Horizonte 31270-901, MG, Brazileniofvet@hotmail.com (E.F.);; 2 Department of Biological Sciences, Kent State University at Stark, North Canton, OH 44720, USA; dqutob@kent.edu; 3 Department of Biology, The Catholic University of America, Washington, DC 20064, USA; 4 Division of Mathematics and Sciences, Walsh University, North Canton, OH 44720, USA; aunderwood@walsh.edu

**Keywords:** SOX3, cancer, apoptosis, epithelial-mesenchymal transition (EMT), invasion, migration, cell cycle, proliferation

## Abstract

Members of the SOX (SRY-related HMG box) family of transcription factors are crucial for embryonic development and cell fate determination. This review investigates the role of SOX3 in cancer, as aberrations in SOX3 expression have been implicated in several cancers, including osteosarcoma, breast, esophageal, endometrial, ovarian, gastric, hepatocellular carcinomas, glioblastoma, and leukemia. These dysregulations modulate key cancer outcomes such as apoptosis, epithelial-mesenchymal transition (EMT), invasion, migration, cell cycle, and proliferation, contributing to cancer development. SOX3 exhibits varied expression patterns correlated with clinicopathological parameters in diverse tumor types. This review aims to elucidate the nuanced role of SOX3 in tumorigenesis, correlating its expression with clinical and pathological characteristics in cancer patients and cellular modelsBy providing a comprehensive exploration of SOX3 involvement in cancer, this review underscores the multifaceted role of SOX3 across distinct tumor types. The complexity uncovered in SOX3 function emphasizes the need for further research to unravel its full potential in cancer therapeutics.

## 1. Introduction

Transcription factors (TFs) are pivotal in shaping cellular identities, directing cell differentiation, and orchestrating complex temporal-spatial gene expression profiles during embryonic development [1,2,3]. The discovery of *SRY* (sex-determining region Y), the founder of the SOX (SRY-related HMG box) protein family of TFs, marked a significant milestone in understanding TF biology in the context of sex determination [4]. The high-mobility group (HMG) box domain within SRY is highly conserved and shared with all 20 SOX protein members and has gained prominence as a versatile DNA-binding motif [5,6,7]. The HMG box DNA-binding domain is composed of three alpha helixes that contain N-terminal and C-terminal nuclear localization sequences, which direct nuclear import. This allows the HMG box to bind and alter DNA architecture by inducing a bend. SOX proteins are modular, with distinct domains inside and outside the HMG box that are essential for the unique functions of the family’s 20 closely related members. Although domains and regions outside the HMG box have been characterized among the SOX proteins, most knowledge about SOX protein activity is associated with the HMG box. This domain drives the binding and bending of DNA, which not only leads to altered expression of target genes but also serves to recruit additional proteins such as p53, Nanog, OCT4, and Wnt/β-catenin, which are required to modulate cellular behavior and fate [8,9,10,11,12]. Like many transcription factors, post-translational modifications and protein–protein interactions impact SOX protein functional outcomes. 

SOX proteins are classified into nine groups based on phylogenetic analysis, each characterized by distinct functions and target genes [13,14]. Among these groups, SOXB1 proteins (SOX1, 2, and 3) play a pivotal role in sustaining stem cell proliferation and maintaining multipotent characteristics. Conversely, other groups, such as SOXB2 (SOX14 and 21), SOXD (SOX5, 6, and 13), and SOXE (SOX8, 9, and 10), function as inhibitors of proliferation, promoting lineage-specific cell identities. In contrast, the SOXC group (SOX4, 11, and 12) drives proliferation and terminal differentiation across diverse lineages [15]. 

In recent years, aberrant expression and function of SOX proteins have emerged as a significant contributor to multiple cancer types. These TFs influence cell differentiation, proliferation, migration, invasion, and metastasis in several tumor types [16,17,18,19,20,21]. The pleiotropic nature of SOX proteins is underscored by their ability to regulate different gene sets in diverse cellular contexts and tissues [14,22]. The complexity of cancer research is compounded by the diversity observed across tumor types, genetic mutations, tumor sites, stages, patient characteristics, treatment responses and outcomes, further challenging the oncology field. Within a single tumor, there is often considerable regional cellular heterogeneity, meaning that different tumor regions have distinct genetic profiles and behavioral characteristics, complicating treatment efficacy [23,24,25,26]. This complexity arises from the intricate interplay of genetic mutations and molecular interactions within the tumor microenvironment [27].

Although there has been considerable progress in understanding the roles of SOX proteins in cancer, identifying specific SOX factors as tumor suppressors or carcinogenic modulators remains challenging. Significantly, SOX proteins within a single family group often exhibit functional overlap or redundancy in the discreet cellular environment. SOX2, a member of the SOX B1 group, has garnered significant attention as a prognostic, diagnostic, and therapeutic target in various cancer types [28,29,30,31,32,33]. However, limited attention has been devoted to another SOX B1 group member, SOX3, leaving its role in cancer relatively unexplored. 

## 2. Functional Implications of OX3 Modifications

SOX3 and other SOX protein activities are known to be modulated by post-translational modifications (PTMs) such as sumoylation, acetylation and phosphorylation, which significantly impact SOX3 function and role in cancer progression [34]. Sumoylation involves the attachment of SUMO (Small Ubiquitin-like MOdifier) proteins to lysine residues within consensus sequences also targeted by ubiquitin on SOX proteins [35]. This modification is mediated by a SUMO E1-activating enzyme, the SUMO-conjugating enzyme Ubc9, and various SUMO E3 ligases. Sumoylation alters SOX3’s stability, subcellular localization, and transcriptional activity [34,36,37,38]. Additionally, acetylation of lysine residues may affect SOX3 interaction with other transcriptional regulators. This modification could increase stability and resistance to degradation, supporting the oncogenic processes.

The interplay between SUMOylation and acetylation may directly regulate SOX function, with acetylation enhancing transcriptional activity and sumoylation reducing transcriptional activity yet maintaining SOX3 abundance intracellularly by protecting it from ubiquitin-mediated degradation [39,40]. This enables SOX3 to participate in complex signaling networks that regulate cancer progression, including pathways involving AKT, NF-κB, and MYC. The presence of phosphorylation-dependent sumoylation motifs (PDSM) further regulates the balance between these modifications, influencing SOX3’s role in cellular signaling [41]. 

These post-translational modifications also impact other SOX proteins involved in cancer. For example, acetylation of SOX2 enhances its stability and transcriptional activity in colorectal cancer, whereas sSUMOylation of SOX10 in melanoma influences its interaction with other transcription factors, affecting gene expression related to melanocyte differentiation and survival [42,43]. In hepatocellular carcinoma, acetylation of SOX17 enhances its tumor-suppressive functions.

Additionally, SUMOylation plays a modulatory role in the nuclear localization of SOX proteins. SUMOylation of SOX9 influences its ability to shuttle between the cytoplasm and the nucleus, thus regulating its transcriptional activity and the expression of target genes involved in tumor progression [44]. Similarly, the sumoylation of SOX10 affects its nuclear localization, impacting its role in gene regulation [45]. Importantly, our group has shown altered nuclear localization of SOX3 in MCF-7 cell lines where SOX3 accumulates in the cytoplasm. 

These studies, which explore the regulatory effects of post-translational modifications on SOX3 and other SOX family proteins, offer a glimpse of the molecular mechanisms driving cancer progression and present potential possibilities for therapeutic intervention. Recent publications include Dutta and Jain [46] on the implications of post-translational modifications in cancer, Li et al. [47] on SUMOylation and ubiquitination crosstalk, and Li et al. [48] on advances in protein modifications in cancer. By understanding these modifications, researchers can develop targeted therapies to disrupt the regulatory networks that promote tumor growth and metastasis [41,49].

SOX3 expression in non-cancerous and cancerous tissues is widespread [50,51,52,53]. This review will discuss the involvement of SOX3 in multiple human neoplasms and present the SOX3 clinical correlation in concert with tumor behavior. Our objective is to shed light on the role of SOX3 in tumorigenesis by examining gene and protein expression patterns in clinical specimens and in vitro and in vivo models across various tumor types. Our review seeks to uncover the multifaceted role of SOX3 in cancer progression, potentially opening new avenues for understanding and targeting this TF in cancer therapeutics.

## 3. SOX3 Involvement and Regulation of Cancer Hallmarks

### 3.1. SOX3 and Cell Death by Apoptosis

Apoptosis is an active, ATP-dependent form of cell death initiated through the activation of proteolytic cascades involving caspases. This process leads to both molecular and morphological alterations within cells, serving as a regulated process to eliminate cells with DNA damage, thus preventing the accumulation of mutations that could potentially lead to cancer [54]. The significance of apoptosis lies in its ability to safeguard the integrity of the cellular environment by orchestrating the removal of compromised cells. A recurrent theme is the potential involvement of SOX3 as either a promoter or inhibitor of apoptosis. This role appears to be contingent upon the specific type of cancer cells under consideration. 

**Figure 1:**
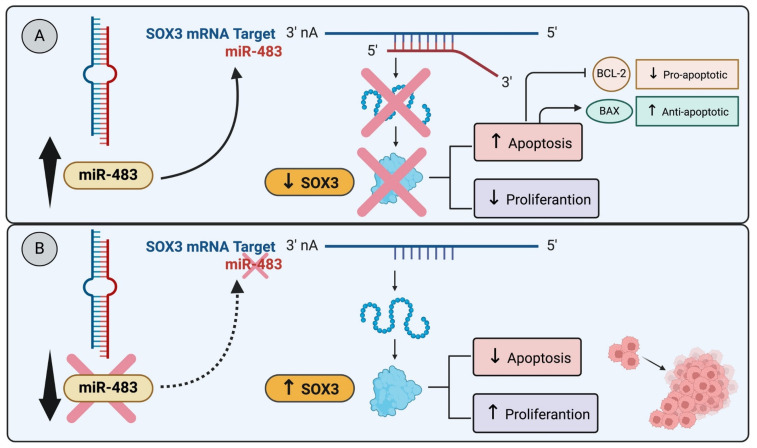
miR-483 modulates SOX3 expression and apoptosis: (**A**) The miR-483 targets SOX3, induces apoptosis, and reduces cell proliferation. The miR-483 transfection into glioma cell lines directly targets SOX3 and downregulates SOX3, enhancing apoptosis in glioma cells [55]. (**B**) The downregulation of miR-483 in breast cancer tissues and luminal A cancer cell lines decreases apoptosis and induces cell proliferation [53].

For example, in breast cancer cell lines MCF-7 and T-47D, both originating from invasive ductal carcinoma luminal A molecular subtype and characterized by differentiated epithelial cells, a study revealed that miR-483 overexpression, targeting SOX3, induced apoptosis leading to a reduction in cell proliferation (Figure 1A). Notably, miR-483 is downregulated in both breast cancer tissues and luminal A breast cancer cell lines [53] (Figure 1B). These findings strongly suggest that SOX3 may play a regulatory role as a blocker of apoptosis, specifically within the context of luminal A breast cancer. Similarly, research by Shujing et al. showed that miR-483 directly targets and downregulates SOX3, which enhances apoptosis in glioma cells [55]. These works illustrate a shared pathway in different types of cancer cells where SOX3 suppression leads to increased apoptosis [55] (Figure 1A).

In contrast, a recent study in breast cancer, utilizing the MDA-MB-231 cell line from invasive ductal carcinoma of the triple negative (TN) molecular subtype and characterized by undifferentiated epithelial cells with mesenchymal morphology, revealed no SOX3 expression. The transfection and expression of SOX3 into these cell lines resulted in the expression of pro-apoptotic markers leading to apoptosis, as detected by Annexin V/PI flow cytometry [56]. These disparate results highlight the complex and context-dependent role of SOX3 in the regulation of apoptosis across different molecular subtypes of breast cancer.

In a study conducted by Guo et al. [50], it was observed that the expression of SOX3 was significantly reduced in osteosarcoma (OS) cell lines. This decrease in SOX3 levels contributed to a higher concentration of OS cells in the G1 phase of interphase and triggered cell apoptosis. This phenomenon was further supported by the observation of lowered Bcl-2 levels, an anti-apoptotic marker, with an increase in the expression of the pro-apoptotic gene Bax [50]. 

Yan et al. [52] investigated SOX3 expression in ovarian carcinoma tissues and SOX3 basal expression in six different ovarian cancer cell lines. SOX3 expression and localization in human ovarian cancer were detected mainly in cell nuclei, whereas normal ovarian tissue samples showed no SOX3 expression. To assess the effect of SOX3 overexpression and silencing in SK-OV-3 (human ovarian cancer cell line with epithelial-like morphology) and SK-OV-3-ip1 (more metastatic) apoptosis, the cells were analyzed using Annexin V/Pi flow cytometry and compared with their control cells. Results revealed a higher percentage of apoptotic cells in SK-OV-3 and SK-OV-3-ip cell lines silenced for SOX3 [52] and a downregulation of apoptosis when SOX3 is overexpressed in these cell lines.

Comprehending the intricacies of apoptosis is essential for establishing precise anti-cancer strategies and discovering innovative therapeutic approaches focused on reinstating apoptotic regulation in cancerous cells. New therapeutic approaches aimed at modulating both the intrinsic and extrinsic pathways of apoptosis, either individually or in combination, hold promise for treating cancer. Oligonucleotides and small molecules designed to mimic the interaction between BH3 proapoptotic members and BCL-2 anti-apoptotic members within the BCL-2 family offer a means of influencing mitochondrial membrane permeability and cytochrome c release. Additionally, targeting cell death receptors (DR) involved in activating the extrinsic apoptosis pathway through external signaling presents a potential mechanistic approach. Agonists capable of binding to DR and initiating cell death signaling show potential in this context [57].

### 3.2. SOX3 and Epithelial-Mesenchymal Transition (EMT)

Epithelial–memenchymal transition (EMT) is a pivotal cellular process with far-reaching implications in oncology. EMT is a reversible program that transforms epithelial cells into mesenchymal cells, involving the loss of adherents junctions and the downregulation of cytokeratins and E-cadherin (epithelial-specific markers), and an increase in mesenchymal markers, such as fibronectin, N-cadherin, and vimentin [58]. In the context of cancer, EMT plays a crucial role in malignant progression by inducing traits such as tumor-initiating properties, motility, dissemination ability, and resistance to chemotherapy. This epigenetic process operates independently of DNA sequence and is orchestrated by EMT-inducing transcription factors (EMT-TFs), such as SNAIL, SLUG, TWIST, and ZEB1/ZEB2 [59]. EMT in carcinoma cells depends on signals from the tumor-associated reactive stroma induced by EMT-TFs, shaping the tumor microenvironment. The detection of EMT-associated protein markers serves as a prognosis indicator of high-grade malignancy in various cancers, including prostate, lung, liver, pancreatic, and breast cancers [60,61,62,63]. 

Qiu et al. [64] identified *SOX3* as a metastasis-associated gene in OS, highlighting its mechanistic connection with the TFs SNAIL1 and MET. *SOX3* was overexpressed in 42 cases of human OS tissues in comparison with non-tumor samples. In addition, MG63 transfected with SOX3 exhibited elevated expression of MET markers, such as N-cadherin and vimentin, and lower expression of epithelial markers, such as E-cadherin and keratin 1, whereas the SOX3 silencing in U2OS cells increases epithelial markers and decreases mesenchymal markers, suggesting SOX3 involvement in EMT in OS cells [64].

**Figure 2:**
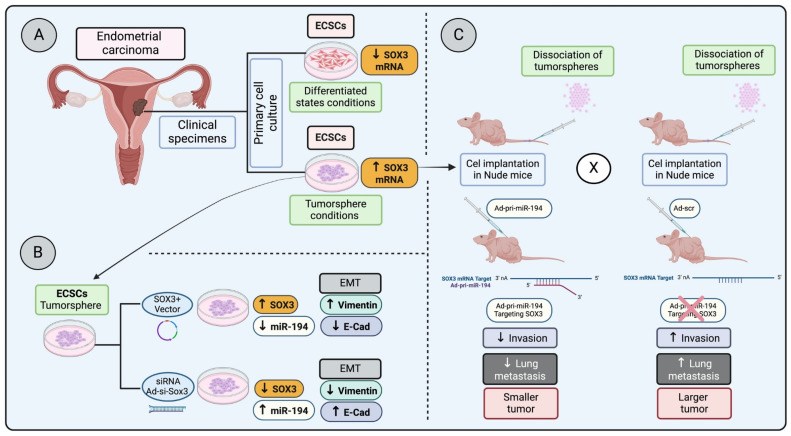
Regulation of SOX3 in endometrial carcinoma progression and metastasis: (**A**) Primary endometrial carcinoma cells cultured in stem cell medium (ECSC) in a differentiated state have a lower SOX3 mRNA expression, whereas ECSC in a tumorsphere condition has a higher SOX3 mRNA expression. (**B**) ECSC in tumorsphere conditions overexpressing SOX3 increases epithelial–mesenchymal transition (EMT) markers, whereas the SOX3 knocked down in the tumorspheres reduces EMT markers. (**C**) Tumor cells dissociated from tumorspheres and injected into nude mice, followed by the injection of Ad-pri- miR-194 (targeting and downregulation SOX3 transcription) or Ad-scr (control), demonstrated that the downregulation of SOX3 decreases invasion, lung metastasis and results in a smaller tumor (low proliferation) [65].

In a study examining endometrial carcinoma stem cells (ECSCs) under both tumorsphere conditions and differentiated states—achieved by removing basic fibroblast growth factor (bFGF)—a significant decrease in SOX3 mRNA expression was observed in differentiated conditions compared to their undifferentiated tumorsphere counterparts (Figure 2A). This research took a further step by injecting dissociated undifferentiated cells from tumorspheres into nude mice, followed by administering Ad-pri-miR-194 targeting SOX3 mRNA (Figure 2C). These results highlight that silencing SOX3 led to reduced invasion and lung metastasis, pointing to SOX3 as a potential marker for ECSCs and suggesting its involvement in invasion, metastasis, and possibly in the regulation of epithelial–mesenchymal transition (EMT) [65] (Figure 2B,C).

However, Silva et al. [66] demonstrate in vitro induction of SOX3 expression results in a decreased expression of the mesenchymal marker N-cadherin (NCAD) and TFs SNAIL, ZEB1, and ZEB2, which play crucial roles in EMT. This aligns with earlier studies that identified elevated levels of SOX3 as key to inhibiting EMT, as seen by the reduced expression of SNAIL in the MCF-7 breast cancer cell line [67]. Moreover, the study observed that MDA-MB-231 cells overexpressing transiently transfected SOX3 exhibited changes in EMT-related TFs and upregulation in E-cadherin (ECAD) gene expression, further substantiating the role of SOX3 in blocking EMT [66].

Understanding and characterizing EMT programs are important in clinical oncology as they contribute to the elevated resistance of mesenchymal carcinoma cells to various treatment regimens, including chemotherapy and immunotherapy.

### 3.3. SOX3 and Cell Invasion and Migration

Epithelial–mesenchymal transition (EMT) is followed by invasion and migration of cancer cells, ultimately leading to metastasis. Notably, an increase in SOX3 expression has been associated with gastric cancer characterized by lymph node metastasis, primary tumor invasion, and a high TNM tumor graduation system [68]. In this study, comprehensive investigations, which included gastric cancer cell lines, a zebrafish in vivo model, as well as clinical samples of patients with gastric cancer, were employed. In vitro experiments demonstrated that silencing *SOX3* reduced the expression of Matrix metalloproteinase-9 (MMP-9). ChIP-PCR confirmed direct transcriptional regulation of the *MMP-9* promoter by SOX3, establishing a pivotal role in the SOX3 transcriptional regulation of MMP-9, a protein crucial for cell invasion and migration processes [68]. 

In osteosarcoma (OS), Guo et al. [50] conducted a SOX3 knockdown in an OS cell line using a Transwell assay, suppressing migration and invasion of OS cells with reduced SOX3 levels. However, this effect was not observed in control cells with basal levels of SOX3 expression [50]. In a parallel study involving ovarian cancer cells, clinical samples, and in vitro approaches, Yan et al. [52] reported that silencing SOX3 in SK-OV-3 cells decreases its ability to migrate and metastasize [52].

Malignant glioblastoma (GBM) is an aggressive cancer characterized by its invasive behavior. In an effort to investigate the SOX3 influence on GBM behavior, Vicentic et al. [51] induced SOX3 overexpression in GBM cell lines. Following the overexpression in U87 and U251 GBM cells, they utilized both Transwell migration and Matrigel assays to assess the cells’ behaviors. The results demonstrated that cells’ increased SOX3 expression leads to enhanced migration and invasion capabilities in vitro [51]. Building on this, Pan et al. [69] discovered that reducing SOX3 expression elevates the migration of the U251 glioblastoma cells, as supported by a wound-healing assay, while not influencing the cellular invasive capabilities at 48 h [69]. Furthermore, research by Shujing et al. [55], which targeted SOX3 with its repressor miR-483, found that downregulating SOX3 suppresses both cell migration and invasion, providing insights into the complex role of SOX3 in GBM behavior.

In esophageal squamous carcinoma samples from 118 patients, both gene and protein expression were evaluated using RT-qPCR coupled with immunohistochemistry, showing no significant correlation in clinical evaluations of primary tumor invasion with SOX3 [70]. Similarly, employing comparable approaches, Feng et al. [71] showed no correlation between SOX3 expression and clinicopathological factors such as tumor emboli and microvascular invasion in hepatocellular carcinoma [71]. Collectively, these observations suggest that SOX3 plays a role in invasion and migration depending on tumor subtype and spatial distribution.

### 3.4. SOX3 Interaction with Cell Cycle Regulators

Cancer is associated with deregulated cell cycle controls. Defects in checkpoints and cyclin-dependent kinase (CDK) activity can drive unrestrained proliferation, increase genomic instability, and contribute to cancer progression and treatment resistance [72]. SOX3 appears to have a role in cell cycle progression. Knockdown experiments with SOX3 resulted in a G1 arrest in OS cells, accompanied by a decrease in the proportion of osteosarcoma MG63 and U2OS cells in the S and G2/M phases [50]. To understand the mechanism of cell cycle alteration in SOX3 knockdown cells, Western blot for Cdc25A, cyclin D1, and PCNA protein quantification indicates a decrease in the expression of these three proteins relative to the control cells [50]. 

**Figure 3:**
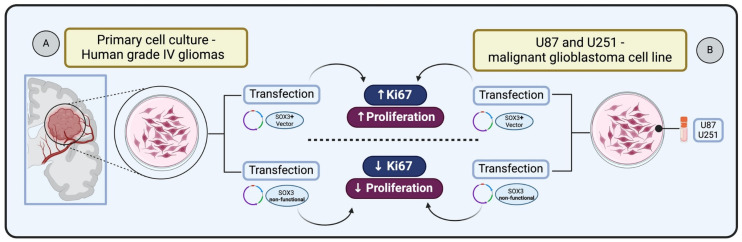
Impact of SOX3 overexpression on glioma cell proliferation: (**A**) Primary glioma cells transfected and overexpressing SOX3 increases Ki67 expression and cell proliferation status [74]. (**B**) Glioblastoma cells transfected and overexpressing SOX3 increase Ki67 and enhance cellular proliferation [51].

SOX3 is known to be involved in central nervous system (CNS) development during embryogenesis. Experiments demonstrating SOX3 gain of function, through cDNAs encoding HMG box of chick Sox3 in expression constructs, followed by electroporation into the neural tube of Hamburger–Hamilton (HH) stage 10 chick embryos, have revealed its capacity to sustain cells as self-renewing progenitors [73]. Holmberg et al. [74] investigated whether SOX3 exhibits this regulatory capacity in glioma cells. They transfected primary cultures derived from human grade IV gliomas with vectors expressing either full-length SOX3 or a dominant negative version of SOX3 (SOX3EnR-Myc). Glioma cells expressing SOX3 showed the presence of cell cycle marker Ki67 within 24 h following transfection. In contrast, glioma cells transfected with the non-functional SOX3 were prompted to exit the cell cycle, resulting in a reduction of cells positive for Ki67 after 24 h. These results indicate that SOX3 can maintain glioma cells in a proliferating state, whereas the active repression of SOX3 target genes causes glioma cells to exit the cell cycle [74] (Figure 3A).

In the context of malignant glioblastoma (GBM), Vicentic et al. [51] identified the upregulation of SOX3 as a key factor in enhancing proliferation. The study involved overexpressing SOX3 in U87 and U251 cell lines transiently transfected with pcDNA3.1/SOX3 construct, leading to a significant increase in proliferation in both cell lines. This effect was confirmed by anti-phosphohistone H3 (pH3) immunostaining, indicating an elevated number of dividing cells and increased expression of the Ki67 marker, which is indicative of enhanced cellular proliferation [51] (Figure 3B).

After conducting functional in vitro experiments involving both the overexpression and silencing of SOX3 in ovarian cancer cell lines (SK-OV-3), Yan et al. [52] assessed the influence of SOX3 on cell proliferation using a CCK8 assay, enabling the quantification of live cells, along with assays to assess colony formation. In ovarian cancer cells, upregulation of SOX3 was found to elevate cell proliferation rate, whereas inhibition reduced cell proliferation. Moreover, the overexpression of SOX3 induced an increased formation of colonies [52]. 

In contrast, when applying a similar overexpression and silencing approach to SOX3 while utilizing the CCK-8 assay to count cells, Shen et al. [68] observed that SOX3 exerted little effect on cell proliferation in gastric cancer cell lines. This discrepancy, again, highlights the context-dependent nature of SOX3 influence on cell proliferation, suggesting that its impact varies across different cancer types.

SOX3, part of the SOXB1 family, is emerging as a critical regulator in the cell cycle and proliferation, interacting with various TFs and regulatory proteins. These interactions likely play roles in crucial phases such as the G1 to S phase transition or cell cycle checkpoint regulation, thus affecting the cell cycle’s timing and progression [75]. Interestingly, SOX3 role is multifaceted; Turchi et al. [76] highlighted its anti-proliferative influence in plasmacytoid dendritic cells (PDC) from primary glioblastoma tumors, presenting a contrast to its previously noted contribution to cancer cell proliferation and tumor progression in different cancers, including gliomas [51,68]. 

This duality in SOX3 function emphasizes the complexity of gene regulation within cancer biology, where SOX3 interactions are pivotal and exceedingly context-dependent. Despite the established significance of interactions with other SOX family members, such as SOX2 and SOX4, with cyclins/CDKs and key signaling pathways like Notch and Wnt, specific documentation regarding SOX3 involvement in these pathways remains scarce [75]. This lack of detailed insight underscores a significant gap in our understanding of the molecular mechanisms through which SOX3 influences the cell cycle and cancer progression, highlighting the need for further research to unravel the intricacies of SOX3 regulatory roles and interactions within the cell cycle and beyond.

## 4. SOX3 Investigation and Clinical Correlation in Different Types of Cancer

SOX3 investigation in different types of cancer: osteosarcoma (OS), breast cancer (BC), gastric cancer (GC), endometrial cancer (EC), esophageal cancer, hepatocellular carcinoma (HCC), lung, ovarian, and acute myeloid leukemia (AML), and its clinical relation with SOX3 expression is summarized and described in Table 1. 

### 4.1. SOX3 in Osteosarcoma

Osteosarcoma (OS) is a rare primary malignant sarcoma in bone, exhibiting osteoid production alongside malignant mesenchymal cells. It ranks as the third most common cancer in adolescence, with an annual incidence of 5.6 cases per million among children under 15. Osteosarcoma typically arises sporadically, with chromosomal abnormalities identified in about 70% of tumor specimens, often involving mutations in tumor-suppressor genes or DNA helicases [86].

In a study involving osteosarcoma patients (*n* = 70), higher gene (RT-qPCR) and protein expression of SOX3 were observed when compared to benign bone lesions [64]. Additionally, in vitro studies using osteosarcoma cell lines indicated that silencing SOX3 expression in osteosarcoma leads to reduced aggressiveness, including proliferation and invasion. Further, there is a positive correlation between SOX3 and genes involved in the epithelial–mesenchymal transition (EMT), suggesting a potential regulatory role. This identifies SOX3 as a potential therapeutic target for metastasis in osteosarcoma [50,64] (Table 1).

### 4.2. SOX3 in Ovarian Cancer

Ovarian cancers (OC) rank as the second leading cause of gynecological cancer-related deaths. Despite advances in treatment, survival rates for stage III and IV epithelial ovarian cancer (EOC) remain at 40% and 20%, respectively. OC treatments involve surgery and platinum-based chemotherapy. However, recurrence is frequent, and current screening methods, including clinical examination and assessment of tumor markers, offer limited benefit in overall survival. Risk factors for OC include age, nulliparity, endometriosis, obesity, and smoking. In addition, hereditary factors, such as BRCA mutations, play a significant role. Although molecular subtyping through immunohistochemistry aids diagnosis and prognosis, the quest for new diagnostic and prognostic markers persists [87].

Positive nuclear accumulation of SOX3 in immunohistochemistry staining was found in human ovarian cancer tissue samples, contrasting with the negative staining observed in control ovarian tissue [52]. This variability in SOX3 expression among human ovarian cancer cell lines highlights its differential behavior depending on the cell type, with metastatic cell lines showing increased SOX3 expression, potentially tying SOX3 to malignant transformations within ovarian tumors. Intriguingly, studies examining the response to chemotherapy drugs like cisplatin found that SOX3 expression was lower in tissues that were more resistant to the drug, suggesting a complex involvement of SOX3 in the progression of ovarian cancer [52] (Table 1).

Expanding upon these insights, Matsumoto et al. [84] explored the impact of anaplastic lymphoma kinase (ALK) overexpression in ovarian cancer [84]. Their research indicates that ALK’s overexpression significantly impacts the biological behavior of ovarian high-grade serous carcinoma (HGSC) and is transcriptionally regulated by the SOXB1 subgroup, which includes SOX3. In HGSC cell lines overexpressing SOX3, ALK expression increased, contributing to the aggressive phenotypic characteristics of HGSC [84]. This body of research not only underscores the pivotal role of SOX3 in ovarian cancer but also connects it with other key molecular players like ALK, offering a better understanding of the molecular underpinnings of ovarian cancer and highlighting potential targets for therapeutic intervention.

### 4.3. SOX3 in Breast Cancer

Breast cancer (BC) stands out as the most prevalent and challenging malignancy affecting women, representing one of the most widespread cancers globally. Its complexity is evident in diverse tumor types characterized by distinct morphology, behavior, and clinical implications. The great heterogeneity in BC poses challenges in understanding and treating the disease. Categorically, BC is divided into three major molecular subtypes based on the positive or negative expression of estrogen or progesterone receptors and human epidermal growth factor 2 (ERBB2 (or human epidermal growth factor receptor 2 (HER2)): hormone receptor-positive/ERBB2 negative (70% of patients), ERBB2 positive (15–20%), and triple negative (tumors lacking all three standard molecular markers; 15%). Breast cancer treatment is intricately linked to the cancer molecular subtype and stage. Early diagnosis significantly enhances survival rates, with a noteworthy 90% chance of survival within 5 years [88,89].

Various scientific investigations have reported changes in SOX genes and/or protein expression in human breast cancer, suggesting that SOX genes contribute significantly to key aspects of breast cancer genesis and progression. In contrast to studies with SOX2, showing it to be associated with aggressive BC and an indicator of poor prognosis [30,76,90], there are limited reports on the role of SOX3 in BC (Table 1). 

Mehta et al. [91] extensively analyzed the transcript profile of SOX family gene expression in breast cancer subtypes [91]. Samples were organized by PAM50 molecular subtype, and patterns of SOX gene expression were determined for 1052 human breast tumors and 94 adjacent standard samples from the TCGA dataset. The analysis revealed altered expression of several SOX genes relative to adjacent normal breast tissue and within the context of the PAM50 molecular subtypes. Notably, SOX3 was excluded from the analysis due to missing or insufficient data (expression values present in >80% of samples) [91] (Table 1).

In a study with invasive ductal carcinoma (IDC) cell lines MCF-7 and MDA-MB-231, Silva et al. [56] reported transcript expression of SOX3 and cytoplasmic localization of SOX3 in MCF-7 cells characterized as an epithelial-like cell and luminal A molecular subtype. In contrast, no SOX3 mRNA or protein was detected in MDA-MB-231 cells, classified as mesenchymal-like and a triple-negative molecular subtype [56]. Following transfection of MDA-MB-231 cells with a SOX3 expression vector, cells upregulated pro-apoptotic genes and increased the apoptotic rate, supported by Annexin V/PI flow cytometry, indicating SOX3 involvement in apoptosis regulation [56]. The study further investigated SOX3 immunohistochemistry localization and quantification in 27 IDC patient samples, along with its correlation with pro-caspase-3 immunoreactivity. Interestingly, positive cases for pro-caspase-3 were negative for SOX3, and the weak staining pattern, with significant SOX3 cytoplasmic localization and a low score (average of 25%), was associated with the cell’s aggressive behavior, indicating downregulation of SOX3 with resistance to apoptosis phenotypes [56] (Table 1). 

In summary, the action of SOX3 in BC, whether as an oncogenic or tumor suppressor, appears to be linked and dependent on the breast cancer histological, molecular, and grade subtype.

### 4.4. SOX3 in Esophageal Cancer

Esophageal cancer, originating in the esophageal epithelium, poses a significant challenge due to low cure rates, especially with late diagnosis [92]. This malignancy is often linked to specific genetic alterations, including mutations in the TP53 tumor suppressor gene, alterations in the CDKN2A gene, and amplifications of the ERBB2 (HER2) oncogene, among others. In esophageal squamous cell carcinoma (ESCC), notable elevations in SOX3 expression were observed compared to non-neoplastic samples [92] (Table 1). 

Clinicopathologic correlation studies indicated that increased SOX3 expression in ESCC is significantly associated with regional lymph node metastasis (RLNM) and advanced TNM staging. This suggests that SOX3 holds promise as a valuable biomarker for prognostic prediction in esophageal cancer and a potential therapeutic target for ESCC [70].

### 4.5. SOX3 in Gastric Cancer (GC)

Gastric cancer (GC) is a complex and unresolved clinical challenge, marked by heterogeneity and particularly high mortality rates in advanced and metastatic stages. This condition remains a significant health concern, with poor overall survival statistics, especially prevalent in Asian and South American countries. Given the variable outcomes associated with the different disease subtypes, the urgency for improved treatment and early detection strategies is evident. Ongoing research explores various emerging therapies and targets in the quest for more effective management strategies [93]. 

In gastric cancer (GC), there is an observed elevation in serum SOX3 expression compared to healthy individuals. This increased SOX3 expression in GC is intricately linked to differentiation, lymph node metastasis, and tumor invasion. The correlation with metastasis is proposed to be influenced by SOX3 positive modulation of matrix metalloproteinase-9 (MMP-9), a key player in cancer cell migration [16,68] (Table 1). Consequently, SOX3 emerges as a promising prognostic factor in GC patients, showing potential oncogenic properties and positioning itself as a candidate for targeted intervention aimed at suppressing cancer progression.

### 4.6. SOX3 in Glioma and Glioblastoma (GBM) 

Glioblastoma (GBM) is recognized as the most aggressive brain tumor due to its rapid and infiltrating growth progression. GBM diagnosis depends on histopathological examination. The molecular subtype of GBM is crucial for both diagnostic accuracy and treatment [94].

In a study led by Vicentic et al. [51], clinical tissue samples and in vitro models were employed to investigate the role of SOX3 in GBM. Immunohistochemical analysis of clinical samples confirmed the presence of SOX3 in the nucleus of all analyzed tumor samples, revealing elevated SOX3 expression in GBM samples compared to non-tumoral brain tissues. Similarly, Yuan et al. [82] and Shujing et al. [55] examined glioma tumor tissues and adjacent normal brain tissues, and SOX3 gene expression was upregulated in glioma tissue clinical specimens compared to that in adjacent normal tissues [55,82] (Table 1).

In a series of in vitro studies, the role of SOX3 in glioma and glioblastoma was investigated, revealing its varied impact on cancer cell behavior [51,55]. Shujing et al. [55] noted that glioma cell lines (LN18 and LN229) exhibited an elevation in SOX3 transcripts when compared to normal brain cell lines (HEB), indicating a potential link between SOX3 expression and glioma pathogenesis. Building on this observation, Vicentic et al. [51] explored SOX3 expression across a broader spectrum of glioblastoma cell lines (U87, U373, U251, A172, and T98). They found that SOX3 expression varied significantly among these lines, and importantly, cells transfected to overexpress SOX3 showed enhanced proliferation, viability, migration, and invasion capabilities [51]. Further extending the investigation into SOX3’s role, Jason et al. [79] used RNA sequencing (RNA-seq) to identify several genes, including SOX3, that were differentially expressed in glioblastoma, correlating with increased tumor invasiveness, malignancy, and a poor prognosis for patients [79] (Table 1).

In a comprehensive exploration of SOX3 in glioblastoma, Pan et al. [69], through the ONCOMINE and CCLE bioinformatic databases, found SOX3 overexpression in glioblastoma tissues compared to normal tissues [69]. This was complemented by a prognostic analysis using the LinkedOmics and GEPIA databases, which presented a positive correlation between higher SOX3 levels and improved overall survival rates in GBM patients, suggesting SOX3 potential as a prognostic biomarker [69]. However, this finding was controversial, as Shujing et al. [55] identified a connection between SOX3 upregulation and poorer patient outcomes in glioma, highlighting the complex role of SOX3 in glioblastoma and glioma pathology (Table 1).

Further investigations into the functional role of SOX3 through in vitro studies using the U251 glioblastoma cell line demonstrated that downregulating SOX3 positively impacted the wound-healing rate, indicating its influence on cell migration [69]. Vicentic et al. [51] furthered this line of inquiry, showing that SOX3 overexpression not only enhanced migration but also increased viability, proliferation, and invasion of glioblastoma cells while reducing autophagy [51]. This effect was particularly pronounced in glioblastoma stem cells and oncospheres, emphasizing SOX3significant role in tumor aggression and stem cell properties [51].

Expanding our understanding of the regulatory mechanisms of SOX3 was a series of in silico experiments and functional assays, revealing that miR-483 and miR-483-3p target SOX3, impacting its expression and thereby affecting tumor cell behavior [55]. This interaction was supported by the findings of Yuan et al. [82], who confirmed the inhibitory effect of miR-483-3p on SOX3 through dual-luciferase reporter assays, linking SOX3 upregulation to increased cell proliferation and anti-apoptotic activity [82] (Table 1). Additionally, the study by Turchi et al. [81] shed light on the RNA-binding protein CELF2’s role as an epigenetic regulator that indirectly represses SOX3, promoting a proliferative tumor cell phenotype and correlating with more aggressive tumor behavior [81]. This relationship between CELF2 and SOX3 underscores the intricate regulatory networks influencing glioblastoma cell dynamics [81].

Additionally, the expression of SOX3 was associated with the presence of the cell cycle marker Ki67, reinforcing its pivotal role in maintaining glioma cells in a proliferative state and promoting malignant behavior in GBM cells [51,74] (Table 1).

Lastly, research by Scuderi et al. [80] introduced a potential therapeutic angle by demonstrating that the chemical compound BX795, a selective inhibitor of PDK1 (pyruvate dehydrogenase kinase1), significantly reduced SOX3 expression in various glioblastoma cell lines, pointing to the targeting of SOX3 pathways as a promising approach to improve disease outcomes [80] (Table 1). Together, these findings paint a complex but enlightening picture of SOX3’s multifaceted role in glioblastoma and glioma, offering valuable insights into its potential as a biomarker and therapeutic target in the fight against these diseases.

### 4.7. SOX3 in Hepatocellular Carcinoma (HCC)

Hepatocellular carcinoma (HCC) stands out as the predominant primary liver malignancy. Risk factors for HCC include chronic liver disease and cirrhosis, with viral hepatitis and excessive alcohol intake ranking as the foremost contributors [95].

A study by Feng et al. [71] delved into SOX3 mRNA expression and protein immunolocalization in HCC tissues compared to non-tumor counterparts. The findings revealed a significant upregulation of SOX3 mRNA and protein expression in HCC tissues compared to adjacent non-tumor regions. Elevated SOX3 expression was associated with advanced tumor progression and worse prognosis in HCC patients. The correlation between SOX3 expression and clinicopathological features further indicated that high SOX3 expression was linked to lower tumor capsule formation, poorer tumor differentiation grades, and worse TNM classification [71] (Table 1). 

The immunolocalization of SOX3 by IHC demonstrated that SOX3 was predominantly localized in the nucleus of tumor cells [71], aligning with its expected role as a TF. These findings underscore the significance of SOX3 as a potential prognostic indicator in HCC and inform treatment strategies. 

### 4.8. SOX3 in Endometrial Carcinoma (EC)

Endometrial carcinoma (EC) is the predominant cancer within the uterine corpus, constituting over 83% of cases. The SOX gene family, particularly SOX2, plays a significant role in carcinogenesis, maintaining cancer stem cell (CSC) pluripotency and regulating cell differentiation, proliferation, and survival. Expression of SOX3 has been identified in EC tissues, correlating with multipotency observed in endometrial tumorspheres cultivated in stem cell medium. These tumorspheres serve as a cancer stem cell model indicative of SOX3 as an ECSC marker. The downregulation of miR-194 was reported as indicative of a poor prognosis in EC. A negative correlation between SOX3 and miR-194 expression with undifferentiated ECSCs was noted, and SOX3 overexpression sustained pluripotency in EC tumorspheres. Elevated SOX3 expression appeared to enhance the epithelial-mesenchymal transition (EMT) process in ECSCs, suggesting its potential to impact clinical outcomes in EC patients [65] (Table 1).

### 4.9. Acute Myeloid Leucemia (AML)

Acute myeloid leukemia (AML) is a complex and heterogeneous disease characterized by rapid cellular proliferation, an aggressive clinical course, variable prognosis, and generally high mortality. A study conducted by Tosic et al. 2018, examined SOX3 gene expression in clinical samples from AML patients and its correlation with clinicopathological aspects. The analysis revealed higher SOX3 expression in 22% of the analyzed AML patients, with a corresponding complete remission rate of 55%. Furthermore, patients with high SOX3 expression exhibited a lower disease-free survival (DFS) than those with low expression, although the difference lacked statistical significance. Overall survival (OS) mirrored the DFS findings, indicating that patients displaying high SOX3 expression had OS of 3 months, not significantly shorter than the 7 months observed in patients with low SOX3 expression [85] (Table 1).

## 5. Conclusions and Future Perspective

**Figure 4:**
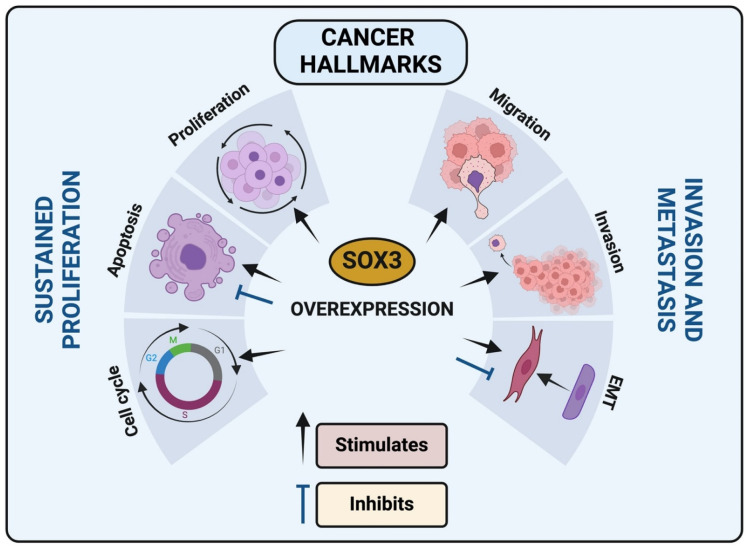
SOX3 impact in cancer hallmarks: SOX3 overexpression in different cancer types can modulate cell cycle, apoptosis, proliferation, migration, invasion and epithelial-mesenchymal transition (EMT).

This comprehensive review describes the multifaceted role of the SOX3 transcription factor within the cancer paradigm. The evidence presented underscores the complexity of SOX3 involvement in the modulation of critical cancer hallmarks, including apoptosis, EMT, invasion, migration, cell cycle regulation, and proliferation (Figure 4). It is apparent that SOX3 function is highly context-dependent, meaning its effects may vary depending on the cellular environment, genetic background, and signaling networks present in different cancer types, and is influenced by the intricate interplay of genetic, epigenetic, and environmental factors. SOX3 may interact differently with other proteins or regulatory elements in distinct cellular contexts, leading to diverse outcomes. SOX3’s dualistic nature as a potential tumor suppressor in certain contexts and a promoter of tumorigenesis in others presents both challenges and opportunities for therapeutic intervention. The correlation of SOX3 expression with clinical outcomes in various cancers emphasizes its potential as a prognostic biomarker and a molecular target for cancer therapy. 

Future perspectives aim to elucidate the complex signaling pathways and interactions involving SOX3 to harness its full potential in the battle against cancer. Revealing the precise molecular mechanisms through which SOX3 influences cancer hallmarks is essential, including deciphering its interactions with other TFs, signaling pathways, and tumor microenvironment cells and components. As a perspective of SOX3 as a therapeutic target, the present review showed that this may include small molecule inhibitors, such as miRNAs and monoclonal antibodies, designed to either inhibit or enhance SOX3 function, tailored to the specific context of its role in various cancers [55,80,81,82]. Adding to that, integration with omics data (genomics, transcriptomics, proteomics), which can help identify novel targets and pathways influenced by SOX3, is essential [55,69,81,82]. Finally, to apply SOX3 in clinical correlation, it is necessary to expand the scope of clinical studies to explore the association between SOX3 expression levels, patient prognosis, and treatment responses across a broader range of cancer types. Such studies should aim to validate SOX3 as a biomarker for cancer diagnosis, prognosis, and prediction of therapeutic response. Investigating the potential of SOX3 as a therapeutic target involves developing and testing novel strategies to modulate its activity. 

## Figures and Tables

**Table 1:** SOX3 in different cancer types and its clinical correlation.

Author	Tumor Type	Specimens/Samples	Methodology/Technique	Main Results/Clinical Correlation
Cui et al., 2019 [53]	Breast	Clinical specimens: 62 patients. Cell lines: MCF-7, SKBR3, LCC2, MDA-MB-453, T-47D, LCC9, and normal human breast cell line MCF-10A	Cell transfection; qRT-PCR; WB; luciferase; cell viability and proliferation (MTT); colony formation and apoptosis detection assay.	The miR-483 inhibitor upregulated the protein level of SOX3. SOX3 expression was negatively correlated with miR-483 expression in breast cancer tissues. The miR-483 could suppress breast cancer cell proliferation and promote cell apoptosis via targeting SOX3.
Silva et al., 2022 [56]	Breast	Cell line: MDA-MB-231	Cell transfection with SOX3 expression vector; immunofluorescence; cell viability and proliferation (MTT); flow cytometry (apoptosis); RT-qPCR.	The apoptotic rate was higher in cells transfected with pEF1-SOX3+ than in controls. MDA-MB-231 transfected with pEF1-SOX3+ showed upregulation of pro-apoptotic CASP3, CASP8, CASP9, and BAX mRNA, contrasting with downregulation of BCL2 anti-apoptotic mRNA, compared to controls.
Silva et al., 2022 [56]	Breast	Clinical specimens: 27 patients with breast invasive ductal carcinoma	Immunohistochemistry	The nuclear expression of the SOX3 protein was detected in 14% of the cases of ductal carcinoma, and the expression of pro-Caspase-3 was positive in 50%. The IHC negative nuclear expression of SOX3 in ductal carcinoma can be related to cells resistant to apoptosis.
Silva et al., 2024 [66]	Breast	Cell lines: MDA-MB-231	Cell transfection with SOX3 expression vector; viability test (MTT); RT-qPCR.	A downregulation in NCAD and an upregulation of ECAD expression, followed by SOX3 protein expression in the triple-negative breast cancer MDA-MB-231 cell line.
Gong et al., 2017 [65]	Endometrial carcinoma	Clinical specimens: 19 endometrial carcinoma patients. Samples (stage IB, *n* = 11; stage IC, *n* = 5; stage IIa, *n* = 3; age = 37–72 years). Primary cell culture with the 19 EC, forming tumorspheres of in vitro experiments. Implantation of tumorsphere cells into mice nude for in vivo experiments.	Constructs for overexpression and silencing SOX3; cell transfection; flow cytometry, RT-qPCR, WB, immunohistochemistry.	SOX3 contributes to endometrial cancer stem cell invasion and suggests that repression of SOX3 by microRNA-194 may have therapeutic potential to suppress endometrial carcinoma metastasis.
Li et al., 2013 [70]	Esophageal squamous	Clinical specimens: 30 patients	RT-qPCR; WB; tissue microarrays; immunohistochemistry.	The expression of SOX3 in esophageal squamous carcinoma (ESCC) was significantly higher than in non-neoplastic samples. SOX3 expression in ESCC significantly correlated with regional lymph node metastasis (RLNM) and advanced TNM. SOX3 may be a valuable biomarker for predicting prognosis and a potential therapeutic target for ESCC.
Cai et al., 2016 [77]	Esophageal squamous	Cell lines: ECA109, SKGT-5, SKTG-4, TE-1, TE-3, TE-8; AND SV40-immortalized non-tumorigenic	Proliferation and cytotoxicity assays (LDH); oncomine; migration and invasion; WB; MMPs activity; RT-qPCR; xenograft model.	SOX3 protein is involved in esophageal squamous carcinoma cell (ESCC) metastasis. SOX3 disruption impaired ESCC cell migration and invasion. Metastasis was significantly inhibited when the SOX3 gene was disrupted by insertional mutagenesis.
Zheng et al., 2017 [78]	Esophageal squamous	Cell lines: TE-1, TE-10, TE-11, EC109, EC9706. Animal model: tumorigenesis and axillary lymph node metastasis in nude mice.	WB; RT-qPCR; invasion; scratch; MTT; tube formation test; ELISA; animal experiments; immunohistochemistry.	SOX3 promotes tumor cell proliferation, migration, and invasion in vitro. SOX3 promotes lymph node metastasis of the tumor in vivo. SOX3 could increase the VEGF-C/D expression in ESCC cells both in vivo and in vitro. The high expression of SOX3 upregulated the expression of VEGF-C and VEGF-D in ESCC and promoted lymph node metastasis.
Shen et al., 2020 [68]	Gastric	Clinical specimens: 60 patients—5 cases of early gastric carcinoma and 55 locally advanced gastric carcinoma cases.	Protein extraction; TMT/iTRAQ labeling; HPLC fractionation; liquid chromatography; WB; ELISA; immunohistochemistry, immunofluorescence.	Serum proteome profiling reveals differential expression of SOX3 protein, between pre- and post-operation for locally advanced gastric cancer. SOX3 is overexpressed in gastric cancer tissues and is associated with poor outcomes for gastric cancer. This study highlights the potentiality of the paired pre- and post-operation serum proteome signature for detecting putative biomarkers for gastric carcinoma and reveals that SOX3 may serve as a candidate molecular marker for the prognosis and outcomes of gastric cancer patients.
Shen et al., 2020 [68]	Gastric	Cell lines: AGS and MKN45 human gastric adenocarcinoma	SOX3 mRNA silencing; invasion assay; xenograft model in zebrafish (zPDX); chromatin immunoprecipitation.	SOX3 promotes gastric cancer cell invasion and migration through MMP9.
Jason et al., 2019 [79]	Glioblastoma	Clinical specimens: glioblastoma (GMB) obtained from patients undergoing surgery was used to obtain primary glioblastoma stem cells (GSCs).	DNA and RNA sequencing; invasion; proliferation; immunoblot assay; flow cytometry; immunohistochemistry.	Identification of several differentially expressed genes, including SOX3, associated with tumor invasiveness, malignancy, and unfavorable prognosis in glioblastoma patients.
Vicentic et al., 2019 [51]	Glioblastoma	Clinical specimens: 13 samples for immunohistochemistry, 27 samples for RT-qPCR, control non-tumoral brain RNAs, and 23 samples for Ambion.	Immunohistochemistry and RT-qPCR	SOX3 expression is higher in glioblastoma samples than in non-tumoral brain tissues. SOX3 protein expression in cell nuclei was observed in all analyzed tumor samples.
Vicentic et al., 2019 [51]	Glioblastoma	Cell lines: U87, U373, U251, A172 and T98; GNS166 and GNS179 (stem cell); GB1 and GB2 (oncopheres).	Transfection and luciferase assay; WB; MTT; immunocytochemistry; Transwell migration and invasion.	Exogenous overexpression of SOX3 enhances proliferation, viability, migration, and invasion of glioblastoma cells. The upregulation of SOX3 was accompanied by improved Hedgehog signaling pathway activity and autophagy suppression in glioblastoma cells. SOX3 expression was elevated in patient-derived glioblastoma stem cells as well as oncospheres derived from glioblastoma cell lines compared to their differentiated counterparts.
Pan et al., 2021 [69]	Glioblastoma	Cell line: U251	Bioinformatic; cell transfection; migration; Transwell invasion assays; RT-qPCR.	Oncomine indicated in the CCLE database shows that SOX3 overexpressed in glioblastoma with a fold change (FC) of 1.184 compared to normal tissue. The LinkedOmics and GEPIA databases showed that increased SOX3 improved overall survival (Logrank *p* = 0.0432). The survival rate of high SOX3 patients is much higher than low SOX3 patients (HR = 0.825), and SOX3 may serve as a prognostic biomarker set for GBM patients. Downregulation of SOX3 increased the wound-healing rate in U251 cells at 48 h, suggesting SOX3 as an antioncogenic. Downregulation of SOX3 has no significant effect on U251 cell invasion.
Scuderi et al., 2021 [80]	Glioblastoma Multiforme	Cell lines: U-138MG, U-87 MG, U-138 and U-87.	Cell viability; RT-qPCR; WB and ELISA.	Treatment of GBM with BX795 (inhibitor of TBK1- TANK-binding kinase) showed a significant reduction in SOX3 gene and protein expression in GBM cells.
Turchi et al., 2023 [81]	Glioblastoma	Cell lines: plasmacytoid dendritic cells (PDC) from primary glioblastoma tumor (GB1, GB5, GB11) and TG6 cells (T lymphoblast).	Bioinformatics; cell transfection with SOX3 specific siRNAs and CELF2-specific shRNA; orthotopic xenografts animal model; RNA sequencing; ChIP sequencing (ChIP-Seq); spheroid formation assays; immunofluorescence; immunohistochemistry; immunohistofluorescence; RNA immunoprecipitation and PCR.	The protein CELF2 acts as an epigenetic regulator in glioma stem cells and can repress the SOX3 gene, promoting a proliferating tumor cell phenotype. CELF2 was found to be a significant point of tumor vulnerability as its repression is sufficient to convert aggressive tumor cells into cells without the ability to form tumors in vivo.
Holmberg et al., 2011 [74]	Glioma (Glioblastoma, Astrocytoma, Oligoastrocytoma)	Clinical specimens: 24 human glioma samples. Cell line: primary cell culture. Animal model: nude mice.	RT-qPCR; WB; immunohistochemistry; immunofluorescence and in situ hybridization.	SOX3 maintains neural cells as self-renewing progenitors, keeping cells on the cell cycle and in a proliferative state.
Shujing et al., 2020 [55]	Glioma	Clinical specimens: 40 patients’ glioma samples and corresponding adjacent tissue samples. Cell lines: gliocyte HEB and glioma cells LN18 and LN229	Bioinformatic; cells transfection with SOX3 expression vector and miR-483; luciferase assay; RT-qPCR; WB and Transwell invasion assay.	The expression level of SOX3 in glioma was significantly higher compared with the normal tissues. SOX3 upregulation was associated with patients predicted poor outcomes. SOX3 mRNA expression was higher in glioma cell lines (LN18 and LN229) than in normal cell lines (HEB). SOX3 is downregulated by miR-483, inhibiting invasion, migration and promoting apoptosis of glioma cells, suggesting that miR- 483 can be a potential target for glioma treatment.
Yuan et al., 2022 [82]	Glioma	Clinical specimens: 50 glioma tumor tissues and adjacent normal brain tissues Cell line: HEB (human normal glial cell) and human glioma cell lines: U87, U251, LN229, and A172.	Bioinformatic; RT-qPCR; WB; cell proliferation assay; 5-ethynyl-2′-deoxyuridine (EdU) assay; flow cytometry; Transwell assay; RNA immunoprecipitation (RIP) assay.	SOX3 gene expression in glioma tissue clinical specimens is upregulated compared to that in adjacent normal tissues. The bioinformatic tool TargetScan predicted SOX3 as the downstream target of miR-483-3p. Functional experiments of dual-luciferase reporter assay confirmed that miR- 483-3p inhibited the activity of the SOX3-WT reporter. In vitro upregulation of SOX3 expression or the inhibition of miR-483-3p expression promotes the proliferation of U87 cells, which was blocked by LINC00662 silencing. Anti-apoptotic protein Bcl-2 expression was inhibited and reversed by co-transfection with SOX3 overexpression plasmids or miR-483-3p inhibitors. LINC00662 triggered tumor-promoting effects in gliomas via modulating the miR-483-3p/SOX3 axis.
Feng et al., 2017 [71]	Hepatocellular carcinoma	Clinical specimens: 50 patients	RT-qPCR; WB; immunohistochemistry.	The mRNA expression of SOX3 is upregulated in hepatocellular carcinoma (HCC) tissues. The recurrence-free survival (RFS) rate of patients with high SOX3 expression was considerably lower than that of patients with basal SOX3 expression. SOX3 overexpression was statistically correlated with less tumor capsule formation, worse degrees of tumor differentiation, and worse TNM classification. Results suggested SOX3 plays an oncogenic role in HCC.
Gure et al., 2000 [83]	Lung	Clinical specimens: 17 patients’ serum with lung cancer and 23 control patients. Cell line SK-LC-13; NCI-H69, 128, 146, 187, 209, 378, 889, 740;	RT-qPCR; Northern blot (NB).	SOX3 mRNA was not detected in serum from normal adult tissues. SOX3 mRNA was detected in 2 out of 10 cell lines. SOX3 is not detectable in normal lung adult tissues. SOX3 expression was detected in 10% of adult lung cancer tissue. All patients with antibodies against SOX3 or SOX21 had higher reactivity against SOX1 and SOX2. The seroreactivity to SOX3 and SOX21 might be secondary to the shared antigenic epitopes located within the highly conserved HMG box of SOX proteins.
Qiu et al., 2017 [64]	Osteosarcoma	Clinical specimens: 42 osteosarcoma tissues; non-tumor samples 42; and bone cysts 28.	RT-qPCR; WB and immunohistochemistry.	SOX3 was overexpressed in most osteosarcoma tissues compared with that in bone cysts. SOX3 expression correlates with Snail1 and E-cadherin in human OS tissues. The mechanistic link among SOX3, Snail1, and EMT indicates SOX3 as a potential therapeutic target for osteosarcoma metastasis.
Qiu et al., 2017 [64]	Osteosarcoma	Cell lines: U2OS, SoSP-M, and MG-63	RT-qPCR; WB; luciferase assay; chromatin immunoprecipitation; cell migration and matrigel invasion; in vivo lung metastasis model; immunohistochemistry.	SOX3 promotes osteosarcoma cell migration invasion and induces EMT upregulating Snail1 expression in osteosarcoma cells.
Guo et al., 2018 [50]	Osteosarcoma	Clinical specimens: 70 patients with primary osteosarcoma and 20 patients with bone cysts	RT-qPCR and WB	Upregulation of SOX3 mRNA and protein expression level in human osteosarcoma tissues. SOX3 acts as an oncogene in osteosarcoma, and SOX3 inhibitors or downstream effectors may be attractive targets for osteosarcoma therapy.
Guo et al., 2018 [50]	Osteosarcoma	Cell lines: MG63 and U2OS human osteosarcoma cells	SOX3 mRNA silencing; WB; cell proliferation; cell cycle; cell migration and invasion assays and cell apoptosis analysis.	SOX3 knockdown in osteosarcoma cells inhibits the proliferation, induces G1 phase arrest, induces apoptosis, suppresses the migration and invasion, suppresses tumor growth in a xenograft mouse model, decreases the EMT-promoting proteins (Twist, Snail, and MMP-9) and increases E-cadherin. SOX3 acts as an oncogene in osteosarcoma, and SOX3 inhibitors or downstream effectors may be interesting targets for osteosarcoma therapy.
Yan et al., 2016 [52]	Ovarian	Clinical specimens: 142 patients with ovarian carcinoma, 28 patients with borderline ovarian cystadenoma, 33 patients with ovarian cystadenoma, and 25 as normal controls.	Immunohistochemistry.	SOX3 immunoreactivity in human ovarian tumor cells was mainly localized to the nuclei. None of the normal ovarian tissue samples were positive for SOX3 expression, whereas SOX3-positive epithelial cells were detected in ovarian cystadenoma, borderline ovarian tumors, and ovarian cancer epithelial tissues. SOX3 expression gradually increased from benign and borderline to malignant ovarian tumors. SOX3 may be involved in the malignant transformation of ovarian tumors and may be used as a supplementary indication in the diagnosis of epithelial ovarian cancer.
Yan et al., 2016 [52]	Ovarian	Cell lines: HO8910; HO8910-pm; SKOV3; SKOV3-ip; ES2; MCV-152; and Moody.	Cell transfection; RT-PCR; WB; cell immunofluorescence; cell proliferation; colony formation; cell migration and invasion; ECM and apoptosis analysis.	SOX3 expression was different in each cell line. SOX3 promotes proliferation, migration, invasionand inhibits the adhesion of ovarian cancer cells. SOX3 inhibits apoptosis of ovarian cancer cells. Overexpression of SOX3 leads to high phosphorylation of pro-metastatic proteins. SOX3 expression was relatively higher in highly metastatic cell lines SKOV3-ip compared to SKOV3 cell line, suggesting that SOX3 may play a key role in cell migration and tumor metastasis.
Matsumoto et al., 2021 [84]	Ovarian Serous Carcinoma	Clinical specimens: 135 cases of ovarian carcinomas. Cell lines: high-grade serous carcinoma (HGSC) cell lines OVSAHO, OVKATE, and OVCAR-3, and ovarian clear cell carcinoma (CCC) cell lines, OVISE, ES-2, OVTOKO, KOC7C, and TOV-21 G.	Immunohistochemistry; in situ hybridization fluorescence; mutation analysis of the ALK and TP53 genes. Cells transfection; RT-qPCR; WB; flow cytometry; spheroid assay; cell counting assay; wound-healing assay and RNA sequencing.	Overexpression of SOX2 or SOX3 enhanced both ALK and ELAVL3 promoter activities, suggesting the existence of ALK/Sox/HuC signaling loops. ALK overexpression was attributed to increased expression of neuroendocrine markers, including synaptophysin, CD56, and B-cell lymphoma 2, in HGSC tissues. These findings suggest that overexpression of full-length ALK may influence the biological behavior of HGSC through cooperation with ELAVL3 and Sox factors, leading to the establishment and maintenance of the aggressive phenotypic characteristics of HGSC. SOX3 expression increased in transfected cells with ALK-overexpressing vector but not in ALK-knockdown cells. Expression of SOX proteins was increased following ALK overexpression, suggesting the existence of a positive feedback loop between ALK and Sox factors. SOX3 induces ALK (anaplastic lymphoma kinase) overexpression in ovarian serous carcinoma.
Tosic et al., 2018 [85]	Acute myeloid leukemia (AML)	Clinical specimens: 50 AML patients with bone marrow and 12 healthy controls (bone marrow donors).	RT-qPCR	SOX3 gene expression was not different from healthy individuals. After the implementation of the “cut-off” value (3.60), 11 (22%) patients with high SOX3 expression were detected. The complete remission rate of patients with high expression of SOX3 was 55%. In the survival analyses, patients with increased expression of SOX3 showed lower disease-free survival (DFS) compared to patients with low expression of SOX3 (4 vs. 14 months). Patients with SOX3 high expression had an overall survival (OS) of only 3 months, but it was not significantly shorter compared to the 7 months found in the patients with low SOX3 expression (Log- Rank = 3.434; *p* = 0.064).

## Abbreviations

BAX	BCL2 associated X—Pro-apoptotic
BCL-2	B-cell lymphoma 2—Regulates apoptosis
BH3	Pro-apoptotic proteins from BCL-2 family
Cdc25A	Cell division cycle 25 A
CDKN2A Gene	Cyclin Dependent Kinase Inhibitor 2A
CELF2	CUGBP Elav-like family member 2—RNA-binding protein
HER2 (ERBB2)	Receptor tyrosine-protein kinase erbB-2
Ki67	nuclear protein associated with cellular proliferation.
Nanog	Transcription factor protein
Notch	notch receptor 1—Type I transmembrane protein
Oct4	Transcription factor protein
p53 (TP53)	Tumor suppressor protein 53
PCNA	Proliferating cell nuclear antigen
SLUG	snail family transcriptional repressor 2
SNAIL	snail family transcriptional repressor 1
TWIST	twist family bHLH transcription factor 1
Wnt	Wnt family member 1
ZEB1	zinc finger E-box binding homeobox 1
ZEB2	zinc finger E-box binding homeobox 2
